# Freezing of Gait in Multiple System Atrophy

**DOI:** 10.3389/fnagi.2022.833287

**Published:** 2022-04-08

**Authors:** Huaguang Yang, Weiyin Vivian Liu, Shanshan Wang, Wenbin Yang, Changsheng Liu, Zhi Wen, Lanhua Hu, Jinxia Guo, Guoguang Fan, Xiaoguang Luo, Yunfei Zha

**Affiliations:** ^1^Department of Radiology, Renmin Hospital of Wuhan University, Wuhan, China; ^2^MR Research, GE Healthcare, Beijing, China; ^3^Department of Radiology, The First Affiliated Hospital of China Medical University, Shenyang, China; ^4^Department of Neurology, The First Affiliated Hospital of South University of Science and Technology, The Second Clinical Medical College of Jinan University, Shenzhen People’s Hospital, Shenzhen, China

**Keywords:** multiple system atrophy, freezing of gait, prevalence, risk factors, epidemiological investigation

## Abstract

**Background and Purpose:**

Freezing of gait (FOG) is a common gait disturbance phenomenon in multiple system atrophy (MSA) patients. The current investigation assessed the incidence FOG in a cross-sectional clinical study, and clinical correlations associated with it.

**Methods:**

Ninety-nine MSA patients from three hospitals in China were consecutively enrolled in the study. Eight patients were subsequently excluded from the analysis due to incomplete information. The prevalence of FOG symptoms in the MSA cohort was determined, and clinical manifestations in MSA patients with and without FOG were assessed.

**Results:**

Of 91 MSA patients, 60 (65.93%) exhibited FOG. The incidence of FOG increased with disease duration and motor severity and was correlated with modified Hoehn and Yahr (H-Y) stages [odds ratio (OR), 0.54; 95% confidence interval (CI), 0.33–3.92], longer disease duration (OR, 0.54, 95% CI, 0.37–0.78), higher Unified Multiple System Atrophy Rating Scale (UMSARS) score (OR, 0.96, 95% CI, 0.93–0.99), MSA-cerebellum subtype (OR, 2.99, 95% CI, 1.22–7.33), levodopa-equivalent dose (LDED) (OR, 0.998, 95% CI, 0.997–1.00), and higher Scale for the Assessment and Rating of Ataxia (SARA) score (OR, 0.80, 95% CI, 0.72–0.89) (logistic regression). Motor dysfunction was significantly positively associated with lower quality of life scores (*p* < 0.01).

**Conclusion:**

FOG is a common symptom in MSA patients and it is correlated with poor quality of life, disease progression and severity, levodopa-equivalent dose, and cerebellum impairment.

## Introduction

Freezing of gait (FOG) is defined as a “brief, episodic absence or dramatic reduction in forward progression of the feet despite the intention to walk.” It frequently occurs in Parkinson’s disease (PD) but is more common in conditions associated with atypical parkinsonism, such as multiple system atrophy (MSA) (Factor, 2008). It is evidently eighty-two patients had from PD, because few studies have observed discovered the differences between the two conditions other than risk factors and incidence rates (Factor, 2008). This brings about the unclear pathogenesis of MSA FOG.

In one previous study the incidence rate of FOG in MSA was 21/28 (75%) (Gurevich and Giladi, 2003). Two other studies reported that 46–54% of patients with parkinsonism-plus syndrome (including MSA patients) had FOG (Giladi et al., 1997; Muller et al., 2002). Freezing symptoms are reportedly to be associated with dementia, incontinence, and tachyphemia. FOG may be more likely to be associated with disabling symptoms in MSA patients than in PD patients due to poorer ataxia in MSA patients (Gellersen et al., 2017). Clarifying FOG risk factors is therefore warranted, to enable clinicians to design appropriate treatment therapies in MSA patients.

The main purposes of the current study were to investigate the incidence rate of FOG in a relatively large Chinese MSA cohort and the clinical factors associated with FOG.

## Patients and Methods

A total of 99 MSA patients were consecutively enrolled in the study from September 2015 to January 2021, and MSA disease diagnoses met the criteria of “possible” and “probable” (Gilman et al., 2008). Eighty-two patients had probable MSA and seventeen had possible MSA (82.83% vs. 17.17%). To exclude other conditions associated with atypical parkinsonism, MSA was diagnosed based on s detailed history of at least 12 months, and a neurological examination by specialists who are experienced in movement disorders. All patients underwent brain magnetic resonance imaging scans, and genomic tests such as that for spinal cerebellar ataxia gene SCA1, 2, 3, 6, and 7. Patients who were younger than 40 years old, had disability during the baseline assessment, were undergoing deep brain stimulation, had serious disease affecting life expectancy in the short term, or did not give consent were excluded. The study was approved by the Ethics Committee of the First Affiliated Hospital of China Medical University, the Shenzhen People’s Hospital, and the Renmin Hospital of Wuhan University. All subjects provided written informed consent before undergoing examinations.

Demographic data including sex, age, educational years, subtype, family history, disease duration, and treatment drugs used were collected by neurologists during face-to-face interviews. Information of pharmacological treatment was collected in detail, and levodopa daily dose and levodopa equivalent daily dose (LDED) were finally accounted (Tomlinson et al., 2010). The Parkinson’s Disease Questionnaire (PDQ)-8 was used to evaluate quality of life (QoL), which contains 8 domains (i.e., mobility, daily living, emotional, stigma, social support, cognitive, communication, and bodily discomfort) (Huang et al., 2011). The Unified Multiple System Atrophy Rating Scale (UMSARS) was used to evaluate motor severity and Hoehn and Yahr (H-Y) stage was used to rating MSA (Stage 1.0: Unilateral involvement only; Stage 1.5: Unilateral and axial involvement; Stage 2.0: Bilateral involvement without impairment of balance; Stage 2.5: Mild bilateral disease with recovery on pull test; Stage 3.0: Mild to moderate bilateral disease; some postural instability; physically independent; Stage 4.0: Severe disability; still able to walk or stand unassisted; Stage 5.0: Wheelchair bound or bedridden unless aided) (Goetz et al., 2004). The Scale for the Assessment and Rating of Ataxia (SARA) was used to evaluate the severity of cerebellar ataxia which contains 8 domains (i.e., gait, stance, sitting, speech disturbance, finger chase, nose-finger, fast alternating hand movements and heel-shin slide) (Matsushima et al., 2017). The Montreal Cognitive Assessment scale (MOCA) scale was used to evaluate cognitive impairment which contains 8 domains (i.e., visuospatial/executive abilities, naming, attention, language, abstraction, memory, and orientation). And mild cognitive impairment was defined as MOCA < 26 points (Dalrymple-Alford et al., 2010). Non-motor symptoms were assessed using the Non-Motor Symptoms Scale (NMSS), which contains 9 domains (i.e., cardiovascular, sleep/fatigue, mood/apathy, perceptual problems/hallucinations, attention/memory, gastrointestinal, urinary, sexual dysfunction, and miscellaneous) (Wang et al., 2009). Severity of depression was evaluated using the Hamilton depression (HAMD) and severity of anxiety was evaluated via the Hamilton Anxiety (HAMA) scales. Patients would be considered as having anxiety or depression if they had eight points or more of HAMA or HAMD scales, respectively (Fava et al., 1982). Considering the prominent autonomic nerve injury symptoms in patients with MSA, this study further evaluated the effects of urinary system function injury and orthostatic hypotension on FOG symptoms in MSA patients. Orthostatic hypotension was defined as a sustained reduction of at least 20 mmHg in systolic BP or 10 mmHg in diastolic BP within 3 min of standing or > 60° head-up tilt (Freeman et al., 2011). Presence of urinary dysfunction is according to standardized protocol of the International Continence Society standard (Abrams et al., 2003).

Freezing episodes were observed by experienced neurologists during hospital visits, and were reported by patients, their family members, or caregivers when they occurred at home or anywhere outside the hospital. Patients were identified as FOG positive, so-called “freezers,” based on item 1 and 3 of the FOG Questionnaire; for example, answering “yes” to the question “Do you feel that your feet get glued to the floor while walking, turning or attempting to initiate walking?” (Giladi et al., 2009). If patients and their family members could not understand the definition of FOG, a description or a possible FOG subtype was determined by neurologists during a hospital visit. Each patient’s answer was confirmed by their relatives or caregivers and by clinical records, to ensure that the accuracy, and severity of freezing symptoms was evaluated using the FOG Questionnaire.

## Statistical Analyses

The Statistical software SPSS 22.0 (SPSS, Inc., Chicago, IL, United States, version 22) was used for all analyses. Demographic and clinical characteristics included age, education, MSA disease duration, dopamine (DA) daily equivalent dose, UMSARS score, HAMD score, and HAMA score. The total scores and each of the NMSS, PDQ-39, SARA, and MOCA domains are presented as mean ± standard deviation. H-Y stages (discontinuous data) are presented as medians. Bivariate analysis, chi-square statistics, or Student’s *t*-test were performed depending on variable type. All categorical variables are presented as numbers or percentages and differences thereof were evaluated via the chi-square test. When comparing the total NMSS and MoCA scores between different groups regarding FOG scores, adjusted for confounding factor of disease duration PDQ-8, intake of DA was performed. A binary logistic regression model with the presence of FOG as the dependent variable was used to investigate potential determinants related to FOG. Only variables with significant differences at the bivariate comparison level were included in step-wise logistic models. The parameters, including: MSA subtypes (MSA-P or MSA-C), disease durations, H-Y stages, LDED, presence of either anxiety, depression or cognitive impairment, PDQ-8, UMSARS, SARA, LDED, as well as the scores of each domain from the PDQ-8, SARA were used as covariables. Hosmer-Lemeshow goodness-of-fit scores were used to assess model fit. In all cases, model fit was higher than 0.8. Multicollinearity was absent from all models. Results are presented as odds ratios (ORs) with 95% confidence intervals (CIs). A two-tailed *p*-value < 0.05 was considered statistically significant (for multiple comparison of chi-square test, *p*-values < 0.017 were considered statistically significant).

## Results

### Incidence of Freezing of Gait

**FIGURE 1:**
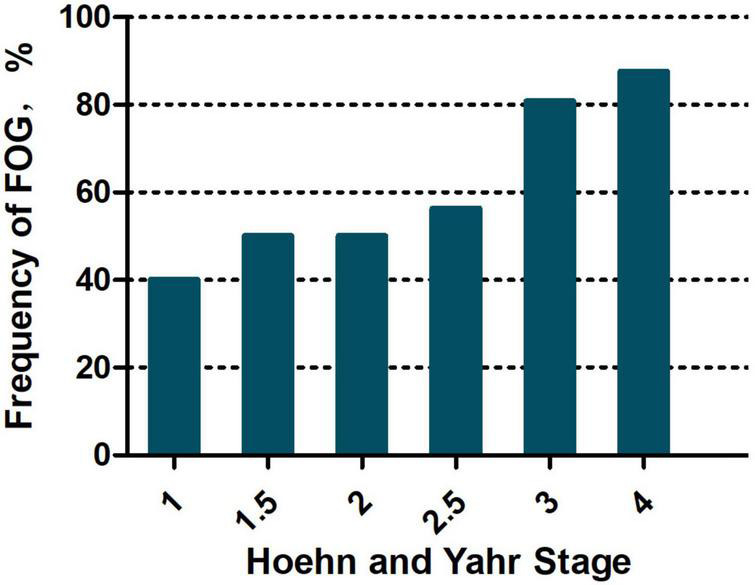
Frequency of FOG based on Hoehn and Yahr stages.

After the exclusion of eight patients from the analysis due to incomplete information, 91 MSA patients (44 male and 47 female) were ultimately included in the analysis, of which 60 (65.93%) were diagnosed with FOG. There was no significant difference in the incidence of FOG between men and women (*p* = 0.655). It was more frequent in patients with the MSA-cerebellum subtype (MSA-C) than those with the MSA-parkinsonian subtype (MSA-P) (75.00% vs. 53.85%, *p* = 0.035). As disease progressed, FOG incidence increased with H-Y stage (stages ≤ 2.5, 51.02%; stage 3, 80.77%; and stages 4, 87.50%; *p* = 0.012, *p* = 0.01, and *p* = 0.57, respectively) (shown in Figure 1). The frequencies of FOG were 48.15% in MSA patients with clinical course < 3 years, 64.29% in MSA patients with courses of 3–5 years, and 81.82% in MSA patients with course > 5 years. There was no significant relationship between age and FOG symptoms (*p* = 0.667) (shown in Table 1).

**TABLE 1:** Incidence of FOG in patients with MSA.

	Groups	Freezers: total sample	Prevalence of FOG	t/χ^2^-value	*P*-value
Total		60:91	65.93%		
Gender^a^	Male	28:44	63.64%	0.200	0.66
	Female	32:47	68.09%		
Age	<65 years old	32:50	64.00%	13.97	0.67
	>65 years old	28:41	68.29%		
MSA subtype	MSA-P	21:39	53.85%	4.44	0.04*
	MSA-C	39:52	75.00%		
Family history	Positive	5:7	71.42%	0.31	0.58
	Negative	51:84	60.71%		
Disease duration	<3 years	11:27	40.74%	6.758	0.01^a^*
	≥3, but <5 years	29:40	72.50%	9.670	0.002^b^*
	≥5 years	20:24	83.33%	0.981	0.32^c^
H&Y stage	<2.5	25:49	51.02%	6.339	0.01^a^*
	3	21:26	80.77%	6.688	0.01^b^*
	4	14:16	87.50%	0.323	0.57^c^

*MSA, multiple system atrophy; MSA-P, MSA-parkinsonian variant; MSA-C, MSA-cerebellar variant; H&Y, Hoehn and Yahr stage; No patient had a stage 5.0 rating.*

*Test a, comparison between patients with a disease duration less than 3 years (or H&Y stage <2.5) and patients with a disease duration between 3 and 5 years (or H&Y stage 3) using a chi-square test.*

*Test b, comparison between patients with a disease duration less than 3 years (or H&Y stage <2.5) and patients with a disease duration greater than 5 years (or H&Y stage 4) using a chi-square test.*

*Test c, comparison between patients with a disease duration between 3 and 5 years (or H&Y stage 3) and patients with a disease duration greater than 5 years (or H&Y stage 4) using a chi-square test.*

For age, gender, MSA subtype and family history, p-values < 0.05 are considered statistically significant, but for Disease duration and H&Y stage, p-values < 0.017 are considered statistically significant (multiple comparison of chi-square test). *, Significant difference.

### Clinical Phenotype of Freezing of Gait

#### Demographic and Clinical Features

There were no significant differences in education years (10.23 ± 3.78 vs. 9.93 ± 2.87, *p* = 0.681), age (62.87 ± 8.09 vs. 64.06 ± 8.01, *p* = 0.23), use of levodopa (*p* = 0.67), dopamine receptor agonist (*p* = 0.06), amantadine (*p* = 0.21), antidepressant (*p* = 0.24), or artane (*p* = 0.14), festination (*p* = 0.14), and falls (*p* = 0.08); MSA duration (4.05 ± 1.85 vs. 2.83 ± 1.50, *p* < 0.01), PDQ-8 scores, UMSARS score (38.03 ± 15.33 vs. 29.73 ± 15.39, *p* = 0.02), H-Y stage (2.88 ± 1.02 vs. 2.39 ± 0.74, *p* = 0.02), and SARA score were significantly higher in freezers than non-freezers (*p* < 0.05); After equivalent dose conversion, MSA patients with FOG symptoms received more daily dose of levodopa than in non-freezer patients (625.00 ± 350.52 vs. 464.87 ± 264.07, *p* = 0.03) (shown in Table 2).

**TABLE 2:** Demographic and clinical features of MSA patients.

	Total (*n* = 91)	Freezers (*n* = 60)	Non-freezers (*n* = 31)	t/χ^2^-value	*p*
**Demographic features**					
Education years (years)	10.02 ± 3.18	10.23 ± 3.78	9.93 ± 2.87	0.017	0.68
Mean age (years)	63.66 ± 8.01	62.87 ± 8.09	64.06 ± 8.01	0.23	0.63
MSA disease duration (years)	3.58 ± 1.82	4.05 ± 1.85	2.83 ± 1.50	-3.492	<0.001*
PDQ-8	13.90 ± 5.76	16.05 ± 5.02	9.74 ± 4.78	352	<0.001*
**Drug usage**					
Levodopa	85 (93.40%)	56 (93.33%)	29 (93.55%)	0.185	0.67
Daily dose of levodopa (mg/day)	570.45 ± 331.07	625.00 ± 350.52	464.87 ± 264.07	674.5	0.03*
Dopamine receptor agonist	41 (45.05%)	30 (50%)	9 (29.03%)	3.669	0.06
Amantadine	15 (16.48%)	12 (20.00%)	3 (9.68%)	1.582	0.21
Antidepressant	71 (78.02%)	49 (81.67%)	22 (70.97%)	1.364	0.24
Artane	29 (31.87%)	16 (16.67%)	13 (41.94%)	2.195	0.14
**Motor severity**					
UMSARS	35.20 ± 15.77	38.03 ± 15.33	29.73 ± 15.39	5.974	0.02*
H&Y stage					
<2.5	49 (53.85%)	25 (41.67%)	24 (77.42%)	6.34	0.012*
3	26 (28.57%)	21 (35.00%)	5 (16.13%)	0.32	0.57
4	16 (17.58%)	14 (23.33%)	2 (0.65%)	6.69	0.01*
**Gait disorders**					
Festination	47 (51.65%)	34 (56.67%)	13 (41.94%)	1.776	0.18
Falls	44 (48.35%)	33 (55.00%)	11 (35.48%)	3.117	0.08
**Cerebellum symptoms**					
SARA	19.09 ± 6.91	21.71 ± 5.72	14.02 ± 6.21	34.82	< 0.001*

MSA, multiple system atrophy; PDQ-8, 8 items Parkinson’s Disease Questionnaire; UMSARS, Unified Multiple System Atrophy Rating Scale; H-Y, Hoehn and Yahr stage; SARA, Scale for the Assessment and Rating of Ataxia; *, Significant difference. p-values < 0.05 are considered statistically significant.

#### Parkinson’s Disease Questionnaire-8, Non-motor Symptoms Scale, Montreal Cognitive Assessment Scale and Scale for the Assessment and Rating of Ataxia

PDQ-8 scores and corresponding scores for MSA in patients with and without FOG are shown in Table 3. Freezers had higher total PDQ-8 scores (16.05 ± 5.02 vs. 9.74 ± 4.78, *p* < 0.001) and higher scores within each domain: mobility (2.77 ± 0.59 vs. 2.13 ± 0.67, *p* < 0.001), daily living (2.25 ± 0.73 vs. 1.10 ± 0.91, *p* < 0.001), stigma (2.08 ± 0.89 vs. 1.26 ± 1.18, *p* < 0.001), social support (1.28 ± 1.29 vs. 0.36 ± 0.80, *p* < 0.001), cognitive (2.25 ± 0.82 vs. 1.48 ± 1.00, *p* < 0.001), communication (1.15 ± 1.20 vs. 0.36 ± 0.75, *p* = 0.001) and bodily discomfort (1.93 ± 1.07 vs. 0.94 ± 1.00, *p* < 0.001) than non-freezers after adjusting for confounding factors including disease duration, UMSARS score, and H-Y stage. MSA patients with FOG symptoms show more serious emotional (depression symptoms) than non-freezers (2.33 ± 1.16 vs. 2.13 ± 1.18, *p* = 0.43).

**TABLE 3:** PDQ-8 of MSA patients with and without FOG.

PDQ-8	Freezers (*n* = 60)	Non-freezers (*n* = 31)	*P*-value
PDQ-8 Total score	16.05 ± 5.02	9.74 ± 4.78	< 0.001*
Mobility	2.77 ± 0.59	2.13 ± 0.67	< 0.001*
Daily living	2.25 ± 0.73	1.10 ± 0.91	< 0.001*
Emotional	2.33 ± 1.16	2.13 ± 1.18	0.43
Stigma	2.08 ± 0.89	1.26 ± 1.18	< 0.001*
Social support	1.28 ± 1.29	0.36 ± 0.80	< 0.001*
Cognitive	2.25 ± 0.82	1.48 ± 1.00	< 0.001*
Communication	1.15 ± 1.20	0.36 ± 0.75	0.001*
Bodily discomfort	1.93 ± 1.07	0.94 ± 1.00	< 0.001*

PDQ-8, 8 items Parkinson’s Disease Questionnaire. * Significant difference.

*p-values < 0.05 are considered statistically significant.*

Total NMSS score and each domain scores for MSA patients with and without FOG are shown in Table 4. Freezers had significant higher total NMS score (59.92 ± 19.12 vs. 52.07 ± 27.31, *p* = 0.11) and each domain: cardiovascular (12.15 ± 4.77 vs. 10.97 ± 4.88, *p* = 0.26), sleep/fatigue (10.7 ± 5.33 vs. 8.03 ± 6.31, *p* = 0.11), mood/apathy (10.47 ± 6.66 vs. 10.16 ± 6.80, *p* = 0.83), perceptual problems/hallucinations (0.87 ± 2.37 vs. 0.32 ± 1.28, *p* = 0.24), attention/memory (5.40 ± 3.89 vs. 5.77 ± 4.65, *p* = 0.69), gastrointestinal (3.93 ± 3.80 vs. 3.32 ± 4.32, *p* = 0.49), urinary (7.77 ± 4.21 vs. 6.55 ± 4.89, *p* = 0.22), sexual dysfunction (5.73 ± 3.20 vs. 4.97 ± 3.70, *p* = 0.31) and miscellaneous (5.68 ± 6.40 vs. 4.19 ± 3.91, *p* = 0.24) scores than non-freezer after adjusting for disease duration, UMSARS score, and H-Y stage.

**TABLE 4:** NMS of MSA patients with and without FOG.

NMS	Freezers (*n* = 60)	Non-freezers (*n* = 31)	*P*-value
**NMSS**			
NMSS total score	59.92 ± 19.12	52.07 ± 27.31	0.11
Cardiovascular	12.15 ± 4.77	10.97 ± 4.88	0.26
Sleep/fatigue	10.7 ± 5.33	8.03 ± 6.31	0.11
Mood/apathy	10.47 ± 6.66	10.16 ± 6.80	0.83
Perceptual problems/hallucinations	0.87 ± 2.37	0.32 ± 1.28	0.24
Attention/memory	5.40 ± 3.89	5.77 ± 4.65	0.69
Gastrointestinal	3.93 ± 3.80	3.32 ± 4.32	0.49
Urinary	7.77 ± 4.21	6.55 ± 4.89	0.22
Sexual dysfunction	5.73 ± 3.20	4.97 ± 3.70	0.31
Miscellaneous	5.68 ± 6.40	4.19 ± 3.91	0.24
**MOCA**			
Moca total score	21.95 ± 6.30	23.87 ± 5.95	0.16
Visuospatial/executive abilities	3.95 ± 1.71	3.16 ± 1.51	0.56
Naming	2.77 ± 0.50	2.68 ± 0.50	0.42
Attention	5.16 ± 0.97	4.67 ± 1.27	0.06
Language	1.90 ± 0.8	1.74 ± 0.93	0.43
Abstraction	1.01 ± 0.68	1.13 ± 0.72	0.46
Memory	1.17 ± 1.12	1.64 ± 1.28	0.70
Orientation	5.45 ± 1.08	5.52 ± 0.81	0.77
**HAMD score**	15.77 ± 9.06	14.77 ± 9.79	0.63
**HAMA score**	13.72 ± 8.35	13.32 ± 10.17	0.84
**Presence of urinary dysfunction**	35 (58.33%)	20 (64.52%)	0.65
**Presence orthostatic hypotension**	27 (45.00%)	11 (35.48%)	0.12

*NMSS, Non-Motor Symptoms Scale; MOCA, Montreal Cognitive Assessment scale. HAMD, Hamilton depression scales; HAMA, Hamilton anxiety scales.*

*p-values < 0.05 are considered statistically significant.*

Urinary system dysfunction (e.g., urinary incontinence) and orthostatic hypotension are core symptoms of autonomic dysfunction in MSA patients. Differences in these two symptoms between freezers and non-freezers in the current study. There were no significant differences in presence of urinary dysfunction and presence orthostatic hypotension (*p* = 0.654 and *p* = 0.116, respectively) (shown in Table 4).

Total MOCA score and each domain scores for MSA patients with and without FOG are shown in Table 4. There were no significant differences in total MOCA score (21.95 ± 6.30 vs. 23.87 ± 5.95, *p* = 0.16) and each domain after adjusting for confounding factors.

Total SARA total scores and corresponding items in MSA patients with and without FOG are shown in Table 5. Total SARA score (21.71 ± 5.72 vs. 14.02 ± 6.21, *p* < 0.001) and each domain: gait (5.22 ± 1.33 vs. 3.84 ± 1.16, *p* < 0.001), stance (3.80 ± 1.18 vs. 2.52 ± 1.09, *p* < 0.001), sitting (2.33 ± 1.02 vs. 1.32 ± 1.17, *p* < 0.001), speech disturbance (2.58 ± 1.17 vs. 1.77 ± 1.31, *p* = 0.003), finger chase (2.11 ± 0.65 vs. 1.15 ± 1.02, *p* < 0.001), nose-finger (1.75 ± 1.03 vs. 1.10 ± 1.03, *p* < 0.005), fast alternating hand movements (2.06 ± 0.66 vs. 1.71 ± 0.80, *p* < 0.03) and heel-shin slide (1.86 ± 1.28 vs. 0.61 ± 1.05, *p* < 0.001). All scores were significantly higher in freezers than non-freezers after adjusting for confounding factors including disease duration, UMSARS score, and H-Y stage.

**TABLE 5:** SARA of MSA patients with and without FOG.

SARA	Freezers (*n* = 60)	Non-freezers (*n* = 31)	*P*-value
SARA total score	21.71 ± 5.72	14.02 ± 6.21	< 0.001*
Gait	5.22 ± 1.33	3.84 ± 1.16	< 0.001*
Stance	3.80 ± 1.18	2.52 ± 1.09	< 0.001*
Sitting	2.33 ± 1.02	1.32 ± 1.17	< 0.001*
Speech disturbance	2.58 ± 1.17	1.77 ± 1.31	0.003*
Finger chase	2.11 ± 0.65	1.15 ± 1.02	< 0.001*
Nose-finger	1.75 ± 1.03	1.10 ± 1.03	0.005*
Fast alternating hand movements	2.06 ± 0.66	1.71 ± 0.80	0.03*
Heel-shin slide	1.86 ± 1.28	0.61 ± 1.05	< 0.001*

The sore of finger chase, Nose-finger, Fast alternating hand movements, Heel-shin slide and Heel-shin slide are the average on the left and right limb. *Significant difference. p-values < 0.05 are considered statistically significant.

### Clinical Factors Predicting Presence of Freezing of Gait

The most significant variables related to FOG in MSA patients were analyzed using a binary logistic regression model based on the forward stepwise method. Disease duration (OR = 0.538, 95% CI = 0.370–0.782, *p* = 0.001), UMSARS score (OR = 0.963, 95% CI = 0.932–0.994, *p* = 0.02), H-Y stage (OR = 0.537, 95% CI = 0.313–3.920, *p* = 0.024), presence of festination (OR = 1.935, 95% CI = 1.180–3.351, *p* = 0.018), phenotype (OR = 2.988, 95% CI = 1.218–7.328, *p* = 0.017), LDED (OR = 0.998, 95% CI = 0.997–1.000, *p* = 0.031), and SARA score (OR = 0.801, 95% CI = 0.724–0.885, *p* = 0.01) were potential determinants of FOG (shown in Table 6).

**TABLE 6:** Correlative clinical factors of FOG in MSA patients.

	OR	95%CI	*P*-value
Age	0.825	0.344–1.981	0.67
Disease duration	0.538	0.370–0.782	0.001*
UMSARS	0.963	0.0.932–0.994	0.02*
H-Y stage	0.537	0.313–3.920	0.02*
Presence of festination	1.935	1.18–3.351	0.02*
Presence of fall	1.415	0.924–2.165	0.11
MSA-C subtype	2.988	1.218–7.328	0.02*
**LDED**	0.998	0.997–1.000	0.03*
**SARA**			
SARA total score	0.801	0.724–0.885	0.00*
Gait	0.832	0.337–2.055	0.69
Stance	0.614	0.206–1.833	0.38
Sitting	1.118	0.573–2.181	0.74
Speech disturbance	0.863	0.539–1.384	0.54
Finger chase	0.427	0.192–0.951	0.06
Nose-finger	1.242	0.66–2.338	0.50
Fast alternating hand movements	1.447	0.593–3.526	0.42
Heel-shin slide	0.568	0.322–1.003	0.05
**Presence of urinary dysfunction**	0.575	0.201–1.648	0.30
**NMSS**	0.983	0.963–1.003	0.10
**HAMD>7**	1.140	0.218–5.978	0.88
**HAMA>7**	0.581	0.104–2.643	0.43
**MOCA<26**	1.016	0.417–2.476	0.97

OR, odds ratio; UMSARS, Unified Multiple System Atrophy Rating Scale; MSA-C, MSA-cerebellar variant; LDED, levodopa daily equivalent dose; SARA, Scale for the Assessment and Rating of Ataxia; NMSS, Non-Motor Symptoms Scale; HAMD, Hamilton depression scales; HAMA, Hamilton anxiety scales; MOCA, Montreal Cognitive Assessment scale. *Significant difference. p-values < 0.05 are considered statistically significant.; No patient had a stage 5.0 rating.

## Discussion

MSA is a rare neurological degenerative disease with an incidence of 7/100,000 (Fanciulli and Wenning, 2015). A total of 91 MSA patients were consecutively recruited into the current study by three hospital centers from north China to south China. This is the first multicenter, cross-sectional study of MSA patients with FOG symptoms, focusing on FOG incidence and clinical correlates of FOG.

The incidence of 65.93% in 91 enrolled MSA patients was slightly lower than that in a previous study (Gurevich and Giladi, 2003); The higher percentage (53.84%) of MSA patients at lower H-Y stages (i.e., < 2.5) may be attributable to a difference in FOG incidence difference. There was no significant difference in FOG incidence between male and female MSA patients (68.09% vs. 63.64%). Patients with FOG had longer disease duration and more advanced disease stage, consistent with previous MSA and PD FOG studies (Gurevich and Giladi, 2003; Ou et al., 2014). As previous MSA and PD studies have strongly suggested, disease progression was a risk factor for FOG.

In the current study, MSA patients with FOG had a lower QoL than those without FOG after adjusting for disease duration, UMSARS score, and H-Y stage. The PDQ-8 is specifically designed to evaluate QoL in patients with PD (Chen et al., 2017). Because there is currently no evaluation scale for QoL in patients with MSA, and due to the similarity of motor and non-motor injuries between MSA patients and PD patients, the PDQ-8 was used to evaluate QoL of MSA patients in the current study, as it has been in previous studies (Zhang et al., 2017, 2019; Raccagni et al., 2019). QoL decreased proportionally with FOG score severity, suggesting that there was a link between FOG and MSA patients’ perceptions of everyday life.

In the present study, freezers took more dopaminergic drugs (dopamine) than non-freezers. In binary regression analysis, levodopa-equivalent dose was a potential dominant factor of FOG. The correlation between dopamine use and FOG symptoms is currently inconclusive. The majority of studies have concluded that striatal dopaminergic denervation plays a critical role in the pathophysiology of FOG in PD given that it occurs most frequently in patients in an “OFF FOG” state who have not received medication yet (Snijders et al., 2016). In some studies, an “ON FOG” state was related to disturbance of cholinergic neurotransmitters but not dopamine (Perez-Lloret et al., 2014). Although dopamine was identified as a risk factor in the present study, suggesting that FOG symptoms in MSA patients were related to the use of DA drugs, caution is needed when drawing this conclusion. First, unlike PD patients, a larger proportion of MSA patients showed no improvement motor function (including gait) after dopamine treatment. Second, “ON FOG” was not differentiated from “OFF FOG” in the current study despite the difference of “ON–OFF FOG” mechanism mentioned in previous studies (Perez-Lloret et al., 2014). The relationship between dopaminergic drugs and FOG may warrant further future research.

Consistent with previous studies, more severe disease progression and more aggravated motor injuries were evident in MSA patients with more frequent FOG (Ou et al., 2014; Gao et al., 2020; Miller et al., 2020). In the current study, the incidence of FOG in MSA patients with FOG (MSA-FOG) at H-Y stages < 2.5 (55.10%) was higher than that in PD patients with FOG (PD-FOG) at H-Y stages 1–2.5 (33.53%) in a previous study (Ou et al., 2014), whereas incidences of MSA-FOG (78.57%) and PD FOG (80.92%) at H-Y stages > 2.5 were similar (Ou et al., 2014). FOG onset occurs earlier in MSA-FOG patients at the early stage of disease, but the onset is similar in the middle and late stages of both MSA and PD. We speculate that compared with PD, MSA progresses faster and motor injuries are more serious, leading to the higher incidence of FOG; but further research is required to confirm this.

The incidence rate of FOG was higher in MSA-C than in those with the MSA-P. Furthermore, cerebellar SARA was a risk factor for FOG onset in the logistic regression model, suggesting that the cerebellum may be involved in the pathogenesis of FOG in MSA patients. The majority of previous studies suggest that the pedunculopontine nucleus but not the cerebellum plays a key role in FOG (Molina et al., 2020, 2021; Chang et al., 2021), because it is mainly involved in the coordination of posture and gait (Gilat et al., 2019). Notably however, in a recent study patient with a damage in the locomotor region of the cerebellum had FOG-like symptoms (Fasano et al., 2017), Two possible explanations for the abnormal involvement of the cerebellum in FOG regulation were proposed: One was damage to specific subregions of the cerebellum in specific locations of the cerebral cortex (e.g., middle frontal cortex) through the “cerebellar–cerebral” loop. Consistent with most previous PD studies indicating that the mid-frontal cortex may be the involved cortical hub, this could explain cognitive loaded FOG (Belluscio et al., 2019; Mi et al., 2019). The other possible explanation was that FOG may be induced by cerebellar injury via abnormal of the “pedunculopontine nucleus–cerebellar” loop coordination or antagonism of input or output information, because there is close connectivity between the pedunculopontine nucleus and the cerebellum with respect to both function and structure, as mentioned in previous reports (Marchal et al., 2019; Holanda et al., 2020). The current study suggests that cerebellum abnormality involvement in FOG pathogenesis may partly explain the higher incidence of FOG in MSA than in primary PD, because MSA patients exhibited more cerebellar damage (Dickson, 2012). Further experiments are needed to confirm whether this effect is pathological or compensatory.

In the present, freezers showed more serious NMS than non-freezers, including more severe cognitive impairment, and more common non-motor symptoms of depression and anxiety after adjusting for disease duration, UMSARS score, and H-Y stage.

Increasing number of studies suggest that cognitive impairment is a risk factor for FOG onset, and cognitive overload is associated with FOG onset (Li et al., 2020; Onder and Ozyurek, 2021). Unlike previous studies (Amboni et al., 2008; Yao et al., 2017), in regression analysis in the present study neither impairment of executive function or visual space function were not related to MSA-FOG in contrast with PD. This does not necessarily mean that FOG incidence is differ in patients with cognitive-mediated MSA and PD. Cognitive evaluation was based on the MOCA scale in present study, in contrast to previous studies investigating the involvement of executive function impairment in PD-FOG regulation, which used other specialized scales (Vitorio et al., 2020; Wu et al., 2020)—this is one limitation of the present study. Future studies need to use a more precise assessment scale to clarify relationships between cognitive impairment and MSA-FOG.

In the current study, freezers had higher Hamilton Depression and Hamilton Anxiety scores than non-freezers but the difference was not significant in multivariate analysis. The current investigation was a cross-sectional study of MSA patients, limiting the possibility of finding baseline features that could predict FOG development as well as parallel progression of FOG with affection problems. The present study only evaluated clinical features not step timing variability (Hausdorff et al., 2003), stride amplitude, or disordered bilateral coordination observed in FOG.

In addition to cerebellar injury, early severe urinary symptoms and orthostatic hypotension are two core clinical features of MSA. Both were more commonly found in freezers than non-freezers in the present study; but in regression analysis there was no relationship between urinary impairment or orthostatic hypotension and MSA-FOG. Freezing symptoms in PD patients included gait freezing and urination freezing (Xu et al., 2020; Lichter et al., 2021). Orthostatic hypotension was a strong independent predictor of falls in another study on the regulation of gait in PD with autonomic dysfunction (Romagnolo et al., 2019). The internal mechanisms associated with the symptoms of autonomic nervous dysfunction and the onset of FOG, and made the relationships between FOG symptoms and autonomic nervous dysfunction remain un explained. Further experiments are needed to identify the internal mechanism s involved.

“The results of the present study confirmed that FOG is a common symptom in MSA patients and is correlated with poor QoL, disease progression and severity, LDED, and cerebellum impairment. These results have some clinical implications. Although the progression of MSA currently cannot be prevented disease modification treatment can be undertaken in the early stage and the optimized the pharmacological treatment can delay MSA progression, QOL is worse in FOG patients than non- freezer patients, and L-dopamine drugs are recommended. The role of cerebellar injury in gait freezing also warrants further attention.”

There current study had several limitations in our study. As an epidemiological study, the samples size was small, partly because MSA is a rare neurodegenerative disease, Future multicenter large-sample studies should be conducted to reduce collinearity between covariables and obtain more reliable results. Some of the assessment scales (i.e., motor, non-motor, and autonomic nervous dysfunction) may not be sufficiently accurate to reflect the symptoms of MSA patients. For example, the MOCA scale is not as accurate as the Frontal Assessment Battery for the assessment of executive function. Assessments of orthostatic hypotension and urinary symptoms cannot replace “autonomic” aspects of scales for outcomes in Parkinson’s disease. Lastly, although spinal cerebellar ataxia genes 1, 2, 3, 6, and 7 are the main subtype in Chinese population, a lack of gene detection of other subtypes will inevitably pollute FOG research in MSA patients, thus it is recommended that future studies use more comprehensive genetic testing to rule out confounding diseases, such as hereditary subacute combined degeneration of the spinal cord. Notably, the study was a phased summary of the MSA-FOG. In a follow-up study including a larger data sample, more comprehensive scales and PD patients with FOG symptoms more stable results would be undoubtedly be obtained.

## Conclusion

FOG is a common symptom in MSA patients, and it is correlated with poorer QoL. FOG is more prevalent in MSA patients with long disease duration and worse motor impairment. Dopaminergic therapy may be protective factor that can improve FOG. The present study indicated that cerebellum impairment was involved in greater FOG severity.

## Data Availability Statement

The original contributions presented in the study are included in the article/supplementary material, further inquiries can be directed to the corresponding author/s.

## Ethics Statement

This study was approved by the Ethics Committee of the First Affiliated Hospital of China Medical University, the Shenzhen People’s Hospital, and the Renmin Hospital of Wuhan University. The patients/participants provided their written informed consent to participate in this study.

## Author Contributions

HY, XL, and YZ contributed to the study conception and design. XL and YZ revised important intellectual content. WL and JG contributed to statistical analysis and language embellishment. HY analyzed, interpreted the data, and wrote the manuscript. YZ, XL, and GF critically revised the manuscript. ZW, LH, WY, and CL contributed to acquisition the data. All authors have approved the submitted version of the manuscript.

## Conflict of Interest

WL and JG were employed by the MR Research, GE Healthcare. The remaining authors declare that the research was conducted in the absence of any commercial or financial relationships that could be construed as a potential conflict of interest.

## Publisher’s Note

All claims expressed in this article are solely those of the authors and do not necessarily represent those of their affiliated organizations, or those of the publisher, the editors and the reviewers. Any product that may be evaluated in this article, or claim that may be made by its manufacturer, is not guaranteed or endorsed by the publisher.
